# Lipotropic activities of aqueous extract of *Vernonia guineensis* Benth. in *Wistar* rats fed high fat diet

**DOI:** 10.1186/s12906-022-03602-4

**Published:** 2022-04-28

**Authors:** Leila Sandra Nnanga, Bruno Dupon Akamba Ambamba, Fils Armand Ella, Damaris Enyegue Mandob, Judith Laure Ngondi

**Affiliations:** 1 Department of Biochemistry, Faculty of Science, University of Yaounde 1, Yaounde, Cameroon; 2 Centre of Research On Medicinal Plants and Traditional Medicine, Institute of Medicinal Research and Medicinal Plants Studies, Ministry of Scientific Research and Innovation, Yaounde, Cameroon; 3 Department of Biological Sciences, Higher Teacher’s Training College, University of Yaounde 1, Yaounde, Cameroon

**Keywords:** Lipotropic agents, NALFD, Cardioprotection, *Vernonia guineensis*

## Abstract

**Background:**

Lipotropic molecules are effective therapeutic targets to counteract non-alcoholic fatty liver disease (NAFLD). Lipotropic compounds are capable of removing fat from the liver and/or manage the reduction of the synthesis or deposition of lipids in the liver. The objective of this study was to evaluate the lipotropic effects of the aqueous extract of leaves of *Vernonia guineensis* (AEVG) on rats fed high fat diet.

**Methods:**

Twenty male rats with an average mass of 235 g were allow acclimatize for seven days, following which they were divided into four groups of five animals each. The test group was treated with high fat diet (HFD) and AEVG at 400 mg/kgBW, while positive control group received HFD and Fenofibrate at 100 mg/kgBW. The normal control group received a normal diet; and the negative control group received HFD. After 14 days of treatment, animals were sacrificed, blood and organs (liver, heart and kidneys), as well as the faeces were collected for the preparation of plasma and homogenates respectively. Some markers of lipid profil (total cholesterol, triglycerides, HDL-c, LDL-c,) and markers of toxicity (AST, ALT, γ-GT, creatinine) were evaluated.

**Results:**

The results obtained showed that a HFD at the hepatic level led to the accumulation of lipids (triglycerides (TG) and total cholesterol (TC)) and had adverse effects on hepatic function by promoting cytolysis. At the plasma level, HFD induced hyperlipidemia. Administration of AEVG at 400 mg/kgBW improved the blood lipid profile and reduced the storage of TG and cholesterol in the liver. AEVG also promoted fecal cholesterol excretion and reduced atherogenic indices which include Total Cholesterol/High-Density Lipoprotein cholesterol (TC/HDL-c) and Low-Density Lipoprotein cholesterol/High-Density Lipoprotein cholesterol (LDL-c/HDL-c). The extract exhibited hepato-protective activity (anticholestasis) and improved glomerular filtration.

**Conclusion:**

These findings suggest that AEVG possesses lipotropic effects confirming its probable use in the management of non-alcoholic fatty liver disease and its cardiometabolic complications. This virtue could be exploited for local pharmaceutical development.

## Background

A significant proportion of the world's human population suffers from an excess of lipids in the liver called non-alcoholic fatty liver disease (NAFLD). NAFLD corresponds to a dysfunction of lipid metabolism and is associated with risk factors for metabolic syndrome in particular visceral obesity, insulin resistance, as well as dyslipidemia (hypertriglyceridemia, increased Low-Density Lipoprotein cholesterol (LDL-c), and decreased plasma High-Density Lipoprotein cholesterol (HDL-c) [1]. At present, there is no specific treatment for NAFLD. However, studies have shown that certain nutrients and bioactive compounds from food such as betaine, choline, some vitamins B (niacin, folate and pantothenic acid) are able to reduce hepatic lipid deposits. These compounds are known as lipotropics which are substances that participate for the removal or decrease the deposition of fat in the liver through their interaction with metabolism of fat [2]. A study on the characterization of the lipotropic potential of 132 products of plant showed that leafy vegetables are a better source of lipotropics, based not only on their content in choline, betaine, magnesium and folate; but also, due to their high content in phenolic acids and flavonoids [3]. Indeed, it has been shown that flavonoids reduce hepatic lipogenesis by fighting against oxidative stress responsible for liver damage and development of NAFLD. These compounds also have beneficial effects on the reduction of lipid absorption (intestine), and visceral obesity (adipose tissue) as well as the increase of β-oxidation of fatty acids (muscles), thereby enabling the fight against the development of NAFLD. On the other hand, animal studies have demonstrated that, they possess hypolipidemic properties to decrease triglycerides and plasma LDL-c levels and increase plasma HDL-c levels [1].

Cameroon is endowed with a diversity of leafy vegetables that are of vital importance because they provide the majority of trace elements, vitamins, and bioactive compounds. It is recognized that local leafy vegetables provide 10 to 100 times more micronutrients than some exotic vegetables such as lettuce [4]. Some local leafy vegetables due to their nutritional values have shown an important role in the prevention of various diet-related chronic diseases in the population. In this context, a major research challenge is to identify local leafy vegetables that can prevent or delay early dysfunctions associated with NALFD. This type of research is crucial to refine and optimize nutritional recommendations and to provide the scientific evidence for the development of new lipotropic functional foods. A systematic review of the scientific literature identified leafy vegetables from the Asteraceae family and the genius Vernonia as potential sources of lipotropic substances [4, 5].

The objective of this study was to evaluate the beneficial effect of the aqueous extract of *Vernonia guineensis* in preventing alterations in lipid metabolism several parts of the human body using an experimental model mimicking a Western diet rich in saturated fatty acids. These will include the gut (decreased lipid absorption), adipose tissue (decrease abdominal fat), plasma (decrease in triglycerides and LDL levels associated with an increase in plasma HDL), and liver (decrease in triglycerides (TG) and total cholesterol (TC).

## Methods

The protocol used in this study complied with relevant institutional, national and international guidelines and legislation required for animal and plant studies.

### Phytochemical investigation of *Vernonia guineensis*

#### Collection of plant material and preparation of aqueous extract

The leaves of *Vernonia guineensis* were collected in September 2019 in Ekangté village in the Nkongsamba II subdivision, in Moungo Division, Littoral Region of Cameroon. The plant was identified by Mr Nana Victor (botanist) and the material collected was identified at the National Herbarium of Cameroon with a voucher number 11133SRF/Cam.

Six hundred (600) g of powdered leaves of *Vernonia guineensis* have been crushed and the powder obtained was introduced in 3 L of boiled distilled water. The mixture obtained was decocted for 40 min and allowed to stay still for 24 h. The supernatant was collected and filtered through Whatman N°1 paper. The filtrate obtained was dried at 60 °C in a ventilated oven for 48 h to obtain the extract.

#### Phytochemical tests

### Qualitative analysis

Qualitative phytochemical analysis was carried out on the aqueous extract of *Vernonia guineensis* using chemical procedures to identify the different classes of secondary metabolites containedin this extract as described for polyphenols by [6]; alkaloids and saponins [7]; flavonoids [8] and tannins [9].

### Determination of total polyphenols content

Polyphenol content was evaluated using the method described by [10]. 30 µL of extract (1 mg/mL) prepared in ethanol was mixed with 1 mL of Folin Ciocalteu (0.2 N) solution. Absorbance was read at 750 nm using a spectrophotometer after 30 min of incubation at 25 °C. Catechin was used as a reference and the results were expressed as microgram equivalence of catechin per gram of extract.

### Determination of total flavonoids content

The quantity of flavonoids in the extract was evaluated according to the procedure described by [6]. One milliliter (1 mL) of sample or standard prepared in ethanol at the concentration of 1 mg/mL was added to 1 mL of AlCl_3_ (10%), 1 mL of potassium acetate (1 M), and 5.6 mL of distilled water. After 30 min of incubation at 25°C, the absorbance was read at 430 nm. Quercetin was used as a standard and the results were expressed as microgram equivalence of Quercetin per gram of extract.

### Animal experimentation and treatment

Ten weeks old male *Wistar* rat strains from the animal house of the Laboratory of Gastroenterology of the University of Yaounde 1 were used. Animals were domesticatedin a cage at room temperature, were regularly fed with a normal diet and had access to water *ad libidum* for 07 days representing acclimatization phase. This study was approved by the Animal Ethics Committee of the Faculty of Sciences, University of Yaounde 1, Cameroon, in accordance with the principles of the *“Guide for the Care and Use of Experimental Animals”* (Committee on Care and Use of Laboratory Animals, 1985) and complied with ARRIVE guidelines.

A high fat diet (HFD) was used as a model to induce lipid metabolism disorders with slight modifications (fat source and lipid content reduction) [11] (Table 1).

**Table 1:** Quantities of the different ingredients needed to prepare 100 g of the normal diet (ND) and high fat diet (HFD)

Ingredients	**ND**	**HFD**
	**Quantity**(g)	**Quantity (g)**
White corn flour, pulped	35.2	35.2
Demi cow's milk powder	15	15
Peanut cake	8	8
Wheat flour	15.2	15.2
Olive oil	7.8	/
Beef tallow	/	15.4
Salt + bone meal	2	2
Polyvitamin	2	2
Cellulose	1	1
Water	13.8	6.2
Total (g)	100	100

#### Experimental protocol

The rats were domesticated under standard conditions of temperature (22 ± 2 °C), relative humidity (50–60%) and 12/12 h light and dark cycle. Animals were randomly divided into 04 groups of 05 each as follows:

- ❖ Group 1: Normal control receiving ND
- ❖ Group 2: Negative control receiving HFD
- ❖ Group 3: Test group receiving HFD and AEVG at a dose of 400 mg/kgBW.
- ❖ Group 4: Positive control receiving HFD and Fenofibrate at a dose of 100 mg/kgBW.

The volume of administration of the extract and reference drug was 5 mL/kgBW. This was administered by esophageal gavage through a tube every morning for 14 days. Parameters such as food intake, rat weight and faeces were assessed every 3 days and the values were averaged.

The weight gain was calculated using the following formula:

$$\mathrm{Weight}\;\mathrm{gain}\;(\%)=\frac{(\mathrm W14-\mathrm W1)}{\mathrm W14}\;\times\;100$$

W1: weight of the rat on day 1.

W14: weight of the rat on day 14.

The ingested energy was obtained by proportionality with reference to the energy intake contained in 100 g of feed.

After that, rats were anesthetized using ketamine/xylazine and euthanized by cervical decapitation. Blood sample was collected from the vena cava, centrifuged for 10 min at 1000 g and plasma was collected. Liver was removed, washed in ice-cold 0.9% saline solution to remove residual blood and then weighed. Faeces were also collected. Liver and faeces were used for lipid extraction. The relative weight (RW) of the organ was calculated using the following formula:

$$Relative\;weight=\frac{\mathrm{Weight}\;of\;\mathrm{organ}}{\mathrm{Weight}\;\mathrm{of}\;\mathrm{rat}}$$

#### Extraction of lipids from faeces and liver

The extraction of lipids from faeces and liver was carried out using the method of [12] with slight modification using the solvent system of hexane-ethanol (3/2; v:v). One (1) g of stool or liver was added to 18 mL of hexane-ethanol solution. The mixture was homogenized for 30 s, and the suspension was filtered through a sintered glass funnel (of medium porosity) equipped with a ball joint for use under pressure. The homogenizer site, funnel, and residue were washed three times with 2 mL of hexane-ethanol solution, re-suspending the residue each time and allowing the solvent to soak for 2 min before applying atmospheric pressure. Non-lipids were removed from the extract by mixing the filtrates for at least 1 min with 12 mL of aqueous sodium sulfate.

### Biochemical analysis

TG and TC contents extracted from faeces, liver and plasma were quantified using the colorimetric method of the SGM italia kit. The HDL-c content was determined using the direct method described by [13], then the estimation of LDL-c and the atherogenic indices TC/HDL-c and LDL-c/HDL-c were calculated. ALT, AST, γ-GT activities and creatinine level in plasma were also measured using the SGM italia kit.

### Statistical analysis

Results are expressed as mean ± standard deviationand as percentage of weight gain. Statistical tests were performed with the SPSS software. Analysis of variance between the different groups was performed using the One-way Analysis Of Variance (ANOVA) test. Tamhane's and Duncan's post hoc comparison tests were used to compare values from the in vivo study. All results with a *p* < 0.05 were considered significant.

## Results

### Phytochemical analysis of AEVG

The results of the qualitative phytochemical analysis of AEVG showed the presence of polyphenols, flavonoids, saponins, condensed and gallic tannins**.** The Table 2 shows the content of phenolic compounds and flavonoids.

**Table 2:** Content in polyphenols and flavonoids

Extract	Polyphenols (µg EC/g of extract)	Flavonoids (µg EQ/g of extract)
AEVG	435.56 ± 10.15	10.39 ± 2.24

*AEVG* aqueous extract of *Vernonia guineensis,* *EC* equivalence of Catechin, *EQ* equivalence of Quercetin

### Effect of AEVG on the evolution of body weight and abdominal fat accumulation

The results showed that feeding with a diet rich in saturated fatty acids increased the weight gain (19%) in the negative control compared to the normal control. However, food intake was not significantly different between these groups. The experimental diet resulted in a significant (*P* < 0.05) increase in abdominal fat (49%) in the negative control compared with the normal control. In Table 3, the administration of the extract did not cause any significant difference in weight gain and food intake compared to the negative control. The group treated with *Vernonia guineensis* significantly (*P* < 0.05) reduced the weight of abdominal fat (11.78 g) and energy intake (254.42 kcal/day) compared to negative control (19.96 g and 278.19 kcal/day respectively). No difference was noted between the AEVG treated group and the positive control (Fenofibrate).

**Table 3:** Effect of AEVG on the evolution of body weight and abdominal fat accumulation

**Groups**	**Weight gain** **(%)**	**Abdominal Fat** **(g)**	**Food intake (g/day)**	**Energy Intake** **(Kcal/day)**
ND + distilled water (Normal Control)	3.10	10.00 ± 2.33	49.66 ± 5,10	207.24 ± 1.07
HFD + distilled water (Negative Control)	3.83	19.96 ± 0.04^b^	59.33 ± 3.29	278.19 ± 2.70
HFD + AEVG (400 mg/kgBW)	-0.69	11.78 ± 4.61^a^	54.26 ± 3.49	254.42 ± 1.97
HFD + Fenofibrate (100 mg/kgBW) (Positive Control)	0.70	9.66 ± 0.47^a^	51.33 ± 13.69	240.68 ± 3.42

*ND* Normal diet, *HFD* high fat diet, *AEVG* aqueous extract of *Vernonia guineensis*

Values were expressed as mean ± standard deviation and as percentage of weight gain

^a^*n* = 5 and ^b^*p* < 0.05; significantly different compared to the negative control and the positive control respectively

### Effect of AEVG on plasma lipoprotein profile and atherogenicity indices

In the plasma, HFD significantly increased TG (35%), TC (58%), LDL-c (77%) levels associated with a significant reduction in plasma HDL-c (-50%) in PC compared to normal control. Similarly, HFD significantly increased the atherogenic indices by (19.38%) for the TC/HDL-c ratio and (14%) for the LDL-c/HDL-c ratio in the negative control compared to normal control. The TC/HDL-c ratio in the negative control was 24.28, well above the threshold of 4.85 and the LDL-c/HDL-c ratio in negative control was 15.57 well above 3.55. According to these results, we found that HFD induced the accumulation of lipids (TG and TC) in the liver and had a pro-atherogenic effect with prognoses of 79% and 89% respectively for the TC/HDL-c and LDL-c/HDL-c index compared to normal control. AEVG treatment prevented significantly (*P* < 0.05) the increase of TG (-39.02 mg/dL) and TC (-27.68 mg/dL) levels compared to negative control. Concomitant administration of AEVG prevented a significant increase (*P* < 0.05) of LDL-c (-21.07 mg/dL) associated with a significant increase (*P* < 0.05) in HDL-c (1.69 mg/dL) compared to negative control. The activity of the extract was more effective than the reference compound (Fenofibrate) at the concentration tested. AEVG also significantly (*P* < 0.05) reduced TC/HDL-c and LDL-c/HDL-c by 9.63 and 5.70 between negative control and normal control 24.28 and 15.57 respectively.

In comparison with negative control, administration of the extract protected the rats against the occurrence of atherosclerosis with the protection percentages of 63.39% and 60.33% respectively for the TC/HDL-c and LDL-c/HDL-c ratios (Table 4).

**Table 4:** Effect of AEVG on plasma lipoprotein profile and atherogenicity indices

**Groups**	**TG** **(mg/dL)**	**TC** **(mg/dL)**		**HDL-c (mg/dL)**	**LDL-c** **(mg/dL)**	**TC/HDL-c**	**LDL-c/ HDL-c**
ND + distilled water (Normal Control)	63.62 ± 8.48^a^	25.88 ± 10.92^a^	3.85 ± 1.05		9.25 ± 1.58^a^	6.90 ± 3.20^a^	3.35 ± 3.27^a^
HFD + distilled water (Negative Control)	98.77 ± 14.52^b^	62.68 ± 16.00^b^	2.56 ± 0.13		40.36 ± 17.15^b^	24.28 ± 5.35^b^	15.57 ± 6.23^b^
HFD + AEVG (400 mg/kgBW)	59.75 ± 30.12^a^	35.00 ± 6.55^a^	4.25 ± 1.42^a^		19.32 ± 10.73^a^	9.63 ± 5.24^a^	5.70 ± 4.88^a^
HFD + Fenofibrate (100 mg/kgBW) (Positive Control)	50.13 ± 8.06^a^	42.47 ± 13.47^a^	4.31 ± 0.66^a^		28.13 ± 14.60	9.78 ± 2.29^a^	6.43 ± 2.67^a^

*ND* Normal diet, *HFD* high fat diet, *AEVG* aqueous extract of *Vernonia guineensis*, *TG* triglycerides, *HDL-c* high density lipoproteins cholesterol, *LDL-c* low density lipoproteins cholesterol

Values were expressed as mean ± standard deviation

^a^*n* = 5 and ^b^*p* < 0.05; significantly different compared to the negative control and the positive control respectively

### Effect of AEVG on hepatic accumulation and faecal excretion of triglycerides and cholesterol

We noted a significant increase in TG (48.65 mg/g liver) and TC (27.31 mg/g liver) levels in the liver of rats in PC compared to normal control. There was no significant difference in fecal TG and TC levels between these two groups**.** Administration of AEVG significantly (*P* < 0.05) reduced hepatic TG (32.48 mg/g liver) and TC (11.99 mg/g liver) levels compared tothe negative control associated with an increase in faecal TC level (72.79 mg/g faeces). However, no difference was noted between AEVG-treated group and positive control (Table 5).

**Table 5:** Effect of AEVG on hepatic accumulation and faecal excretion of triglycerides and cholesterol

Groups	Liver		Faeces	
	**TG** **(mg/g of liver)**	**TC** **( mg/g of liver)**	**TG** **( mg/g of faeces)**	**TC** **( mg/g of faeces)**
**ND + distilled water (Normal Control)**	9.73 ± 1.77^ab^	20.06 ± 1.68^b^	110.46 ± 12.08^ab^	57.13 ± 15.78^b^
**HFD + distilled water ( Negative Control)**	48.65 ± 8.81^b^	27.31 ± 9.94^b^	191.64 ± 6.77^b^	46.41 ± 6.25^b^
**HFD + AEVG** **(400 mg/kgBW)**	32.48 ± 9.98^a,b^	11.99 ± 2.03^a^	198.43 ± 5.82^b^	72.79 ± 7.41^a^
**HFD + Fenofibrate** **(100 mg/kgBW)** **(Positive Control)**	16.10 ± 13.83^a^	4.97 ± 2.19^a^	257.90 ± 20.07^a^	63.34 ± 2.23^a^

*ND* Normal diet, *HFD* high fat diet, *AEVG* aqueous extract of *Vernonia guineensis*, *TG* triglycerides, *TC* total cholesterol

Values were expressed as mean ± standard deviation

^a^*n* = 5 and ^b^*p* < 0.05; significantly different compared to the negative control and the positive control respectively

### Effect of the extract on the hepatic function

There was a significant difference (*P* < 0.05) between the relative liver weight of rats in negative control (0.035) and normal control (0.025). Concomitant administration of AEVG significantly (*P* < 0.05) decreased the relative liver weight (0.024) compared to negative control (0.035). Moreover, HFD significantly (*P* < 0.05) increased the activity of the liver cytolysis marker enzymes AST and ALT (87.64 U/L and 98.78 U/L) compared to normal control (69.97 U/L and 80.22 U/L) respectively. Furthermore, HFD significantly (*P* < 0.05) increased γ-GT activity (2.65 U/L) in negative control compared to normal control (0.98 U/L). Although there was no significant difference in relative weight of kidney in the different groups. HFD significantly (*P* < 0.05) increased the level of creatinine (15.9 mg/dL) in normal control (6.14 mg/dL). Administration of AEVG did not significantly influence ALT activity; but it significantly (*P* < 0.05) reduced AST activity (1.54 U/L) compared to negative control (2.56 U/L).

Although there was no significant difference in kidney relative weight between the different groups, co-administration of AEVG significantly reduced creatinine level (6.43 mg/dL) compared to negative control (15.90 mg/dL). The activity of the extract was more rebooted than the positive control (Fenofibrate) (Table 6).

**Table 6:** Effect of the extract on the hepatic function

Groups	RW Liver	RW Kidneys	AST (U/L)	ALT (U/L)	γ-GT (U/L)
ND + distilled water (Normal Control)	0.0270 ± 0.005^b^	0.0072 ± 0.00006	69.97 ± 13.36^a^	80.22 ± 12.71^a^	0.98 ± 0.23^a^
HFD + distilled water ( Negative Control)	0.035 ± 0.0057^b^	0.0081 ± 0.00025	87.64 ± 15.17	98.78 ± 10.26	2.65 ± 1.70
HFD + AEVG (400 mg/kgBW)	0.024 ± 0.0023^a,b^	0.0074 ± 0.00082	62.96 ± 33.45	110.34 ± 19.56	1.64 ± 1.14
HFD + Fenofibrate (100 mg/kgBW) (Positive Control)	0.018 ± 0.002 ^a^	0.0062 ± 0.00065^a^	76.12 ± 18.05	125.13 ± 13.80	1.84 ± 0.75

*ND* Normal diet, *HFD* high fat diet, *AEVG* aqueous extract of *Vernonia guineensis*, *RW* relative weight, *AST* aspartate aminotransferase, *ALT* alanine aminotransferase, *γ-GT* gammaglutamyl transferase

Values were expressed as mean ± standard deviation

^a^*n* = 5 and ^b^*p* < 0.05; significantly different compared to the negative control and the positive control respectively

## Discussion

Non-alcoholic fatty liver disease (NAFLD) is triggered by excessive hepatic triglycerides synthesis using *de novo* lipogenesis of fatty acids derived from adipose tissue, and endocytosed remnants of triglyceride rich lipoproteins [14]. The objective of this study was to evaluate the effect of the aqueous extract of *Vernonia guineensis* in preventing alterations in lipid metabolism in the intestine, adipose tissue, plasma and liver using an experimental model mimicking a Western diet rich in saturated fatty acids.

The phytochemical study of the extract revealed the presence of some metabolites such as polyphenols, flavonoids, saponin, gallic and condensed tannins. However, the presence or not of a certain bioactive compounds in a plant material depends on the specie, the part used and the region where it is found [15, 16]. These compounds have lipotropic activities. A lipotropic compound increases the exportation of hepatic fat that decreases hepatic lipids and increases adipose and serum lipids.

After feeding the animals with HFD, there is a significant increase in abdominal fat weight in the negative control. High-fat diet leads to increased abdominal fat weight in rats [17]. This could be explained by the fact that the rats ingested more energy (25%) and recorded a significant increase in plasmatic and hepatic TG levels because the rats were fed on a HFD containing TG. In normal situation, insulin has an inhibitory effect on hormone-sensitive lipase in the adipose tissue (AT) leading to insulin resistance (IR). This effect was inhibited, resulting in an excessive degradation of TG in AT with an increased release of free fatty acids (FFAs) towards the liver, which could explain a high TG level in the liver [18]. It was observed that plasma and hepatic TC were significantly increased in the positive control [19].The results obtained could be explained by the presence of saturated fatty acids and cholesterol in the high fat diet which are the precursors of endogenous cholesterol biosynthesis [20].

To explore lipotropic mechanisms, the effect of AEVG was evaluated on lipid digestion and absorption. For this purpose, the fecal TG levels as well as the fecal TC level were evaluated. In comparison with negative control, administration of AEVG to rats receiving HFD resulted in a significant increase of the level of TC in faeces. This result is due to the presence of bioactive compounds (polyphenols and flavonoids) in AEVG that would inhibit the intestinal cholesterol transporter (NPC1L1), increasing dietary and biliary cholesterol in the feces [21]; and saponins that can form insoluble complexes with dietary cholesterol and prevent cholesterol absorption [22].

Exploring lipogenesis and hepatic lipid storage, the concomitant administration of AEVG decreased plasmatic and hepatic TG values compared to the negative control. AEVG contains polyphenols and saponins which are able to inhibit TG synthesis in the liver [23]. According to literature, polyphenols from tea have the capacity to inhibit lipogenesis [1]. For cholesterol, there was a significant decrease in hepatic and plasma TC levels in the AEVG-treated group compared to negative control through inhibition of HMG-CoA reductase by the saponins [24] and polyphenols which breaks the enterohepatic cycle of cholesterol, thus leading to a purification of blood cholesterol for the formation of bile salts [21]. Regarding the effect of AEVG on cholesterol fractions, administration of the extract significantly reduced plasma LDL-c level and increased plasma HDL-c level compared to the negative control. Flavonoids are able to decrease the expression of genes that express low density lipoproteins (LDL) [25]. These bioactive molecules can induce trans-intestinal excretion of cholesterol which enable the direct elimination of LDL-c from blood in the intestinal lumen [26], facilitate the internalization and degradation of IDL in the liver thus preventing their transformation into LDL. The increase of HDL-c in the trial group in comparison with negative control could be due to the polyphenols of AEVG that promote the process of reverse cholesterol transport through the ATP-binding cassette A1 (ABCA1) pathway [27]. Furthermore, low cholesterol level in the liver triggers an increase in LDL receptors expression in hepatocytes and the subsequent removal of LDL from the circulation, which could explain the low level of plasma LDL in the trial group [28].

Exploring the effect of the extract on the development of atherosclerosis, AEVG significantly reduced the ratio of cardiac risk factors (TC/HDL-c and LDL-c/HDL-c) compared to the negative control [29]. These results reflect the anti-atherogenic effect of AEVG, suggesting its protective effect mediated phytochemical constituents in AEVG which reduce LDL-c and increase HDL-c. In addition, it has been reported that HDL-c is potentially able to inhibit LDL oxidation to prevent the development of atherosclerosis [30].

In this study, to evaluate the effect of AEVG on liver and kidney toxicity, the relative weights of livers and kidneys were calculated and then the activities of transaminases (AST, ALT, γ-GT) and plasma creatinine were evaluated. The results obtained show a significant decrease in relative rat liver weight. The activities of transaminases (AST and ALT) increased in the negative control. HFD promotes fat accumulation in the liver and leads to cellular dysfunction and liver damage that induce hepatic cytolysis causing the release of these intracellular enzymes into the bloodstream and decrease the secretion of bile acids [31]. The decrease of relative rat liver weight, plasma AST and γ-GT activities indicate a possible hepatoprotective potential of this extract. However, we observed a slight increase in plasma ALT activity compared to the negative control which showed reduced toxicity of AEVG due to the presence of saponins, which possess a slight hepatotoxicity [32].

Finally, with the influence of the extract on the nephrotoxicity marker, a significant increase in creatinine level in the plasma of the negative control was recorded. Indeed, a high level of creatinine in plasma is characteristic of a glomerular filtration defect. The administration of the extract led to a significant decrease in creatinine level. This effect is due to the presence of phenolic compounds which by their antioxidant role would limit the effect of pro-oxidants in the kidneys [33]. This activity relates the increase in clearance of these wastes with improvement of glomerular filtration.

## Conclusion

In summary, the study demonstrated that AEVG exhibit lipotrophic potential in rats fed HFD through the decrease of hepatic triglycerides and cholesterol levels in the liver and blood. AEVG ameliorated cardiometabolic risks factors. These findings suggest that AEVG may be a potential candidate to prevent accumulation of fatty in the liver.

## Data Availability

The data and material of this study are available on request from the corresponding author.

## Abbreviations

AEVG: Aqueous Extract of *Vernonia **guineensis*
ALT: Alanine Amino Transferase
AST: Aspartate Amino Transferase
HFD: High Fat Diet
FFA: Free Fatty Acids
IR: Insulin Resistance
HDL-c: High Density Lipoprotein Cholesterol
LDL-c: Low Density Lipoprotein cholesterol
NAFLD: Non-alcoholic Fatty Liver Disease
ND: Normal Diet
TC: Total Cholesterol
TG: Triglycerides
TICE: Trans-intestinal Excretion of Cholesterol
VLDL: Very Low Density Lipoprotein
γ-GT: Gamma Glutamyl Transferase
